# The importance of acute kidney injury in suspected community acquired infection

**DOI:** 10.1371/journal.pone.0216412

**Published:** 2019-05-07

**Authors:** James Tollitt, Nicola Bennett, Denise Darby, Emma Flanagan, Paul Chadwick, Smeeta Sinha, Philip A. Kalra, James Ritchie, Dimitrios Poulikakos

**Affiliations:** 1 Renal Department, Salford Royal NHS Trust, Salford, United Kingdom; 2 University of Manchester, Oxford Road, Manchester, United Kingdom; 3 Pathology Department, Salford Royal NHS Trust, Salford, United Kingdom; 4 Informatics Department, Salford Royal NHS Trust, Salford, United Kingdom; 5 AKI Lead for Greater Manchester and Eastern Cheshire Strategic Clinical Networks, Manchester, United Kingdom; National Yang-Ming University, TAIWAN

## Abstract

**Background:**

Most sepsis and acute kidney injury (AKI) cases are community acquired (CA). The aim of this study was to evaluate the characteristics of suspected community acquired infection (sCA-I) and CA-AKI and their impact upon patient outcomes.

**Methods:**

All adult creatinine blood tests from non-elective, non-dialysis attendances to a single centre over a 29-month period were analysed retrospectively. We defined sCA-I and CA-AKI cases as antibiotic prescription and AKI alert within 48 hours of attendance respectively. Binary logistic regression models were created to determine associations with 30-day mortality, intensive care unit (ICU) admission and length of stay (LOS) dichotomised at median.

**Results:**

Of 61,471 attendances 28.1% and 5.7% suffered sCA-I or CA-AKI in isolation respectively, 3.4% suffered both. sCA-I was present in 58.8% of CA-AKI cases and CA-AKI was present in 11.9% of CA-I cases. The combination of sCA-I and CA-AKI was associated with a higher risk for all outcomes compared to sCA-I or CA-AKI in isolation. The 30-day mortality was 8.1%, 11.8% and 26.2% in patients with sCA-I, CA-AKI and when sCA-I and CA-AKI occurred in combination respectively. The adjusted odds ratios (OR) and 95% confidence intervals (CI) for 30-day mortality, ICU admission and LOS for sCA-I combined with CA-AKI stage 1 were OR 6.09:CI: 5.21–7.12, OR 12.52 CI: 10.54–14.88 and OR 8.97 CI: 7.62–10.56, respectively, and for combined sCA-I and CA-AKI stage 3 were OR 9.23 CI: 6.91–12.33, OR 29.26 CI: 22.46–38.18 and OR 9.48 CI: 6.82–13.18 respectively.

**Conclusion:**

The combination of sCA-I and CA-AKI is associated with worse outcomes.

## Introduction

Sepsis is a major health problem associated with high morbidity and mortality. It is estimated that 70% of cases arise within the community [1]. Sepsis is defined as life threatening organ dysfunction caused by a dysregulated host response to infection. The organ dysfunction can be depicted by an increase in the Sequential (Sepsis Related) Organ Failure Assessment score where renal is one of the five systems assessed [2]. Guidance from the National Institute for Health and Care Excellence suggests that the presence of Acute Kidney Injury (AKI) during the assessment of suspected infection in acute hospital settings setting identifies individuals with high risk of severe sepsis whom should then urgently receive antibiotics and the sepsis care bundle [2].

AKI is common and associated with poor outcomes [3]. The majority of AKI is community acquired (CA-AKI) whereby the insult to the kidney occurs before admission to hospital but is detected in the first hospital assessment when creatinine level can be measured [4]. The reported incidence of CA-AKI varies due to differing populations, hospital settings, definition of AKI and time between triggering blood test and hospital admission [5]. Stucker et al demonstrated an incidence of CA-AKI in 4% of emergency attendances to a hospital in a European urban population using the last previous available creatinine value within the preceding 12 months as referent creatinine to define CA-AKI [6]. This incidence is similar to that in a UK population which demonstrated a CA-AKI incidence rate of 4.3% when applying the NHS AKI algorithm. This algorithm uses either the lowest creatinine value during the preceding week and if not available the median creatinine value over the preceding 12 months [7]. CA-AKI is largely accepted as AKI within 48 hours of admission [8].

During recent years concerted efforts to tackle AKI have demonstrated reduced incidence, improved outcomes and reduced mortality with regards hospital acquired AKI [9–11] however the burden and outcomes of CA-AKI remain largely unaffected. The aetiology and characteristics of CA-AKI remain incompletely understood due to limitations in ascertaining and coding the cause of AKI at the point of hospital admission. Improved characterisation of CA-AKI would enable the design of effective interventions to improve outcomes in particular in cases with infection associated AKI which is likely to be a major contributor to CA-AKI cases.

The aim of this study was to determine the prevalence of sCA-I and CA-AKI and the impact of their coexistence on 30-day all-cause mortality, intensive care unit (ICU) admission and length of stay (LOS). The study also aimed to examine these associations separately in care home residents covered from a single provider because of the known increased vulnerability to both infection and AKI.

## Materials and methods

### Ethical statement

Approval by ethical committee was not sought as the data was collected as part of a collaborative quality improvement project at Salford Royal NHS Trust. This aimed to review and improve the recognition and management of AKI [11].

### Study methods

All non-elective hospital attendances to a single tertiary centre who underwent phlebotomy for creatinine measurement were recorded between 1/1/2015 and 31/5/2017. The centre has a catchment area of 250,000 people. Patients with end stage renal disease were excluded. Only patients registered with a local general practice were included so that care home patients are accurately represented and the data captured secondary care attendances and not tertiary. Care home patients in this area are covered by a hospital managed, general practitioner and geriatrician delivered service. In this way most care home patients can be identified by the code of the primary care provider. Demographic data were garnered from electronic patient records. Patients were divided into 4 groups: sCA-I, CA-AKI, sCA-I and CA-AKI and patients with neither suspected infection or AKI. This latter group represented referent group in the analysis. Patient data was anonymised prior to researchers gaining access to it.

AKI was determined using the NHS AKI algorithm which uses change in creatinine from baseline to determine AKI stage [12]. In brief this classification is based upon the KDIGO AKI staging system [13] but uses only changes in creatinine and not urine output. AKI3 is alerted when the creatinine is greater than 3x reference creatinine value or >1.5x reference value and greater than 354 μmol/L. AKI2 is alerted where the creatinine value is between 2-3x reference value and AKI1 is alerted when the creatinine is 1.5-2x reference value or has increased by 26 μmol within 48hrs. This AKI staging mechanism has been externally validated with a high degree of sensitivity and specificity [14]. AKI which triggered an AKI alert and necessitated haemodialysis or hemofiltration within 48hrs, as defined by procedure coding, was defined as CA-AKI stage 3.

sCA-I was defined as an electronic hospital prescription of any antibiotic within 48 hours of attendance. Prescribed antibiotic was used as a surrogate for suspected infection [15–17]. CA-AKI was defined as an AKI of any stage within 48 hours from time of hospital attendance [8]. Our centre has been undertaking quality improvement work in the emergency departments and has been auditing compliance with antibiotic stewardship on a monthly basis [18]. This has demonstrated excellent compliance (mean 96% in emergency assessment unit and 99% in the emergency department) with antibiotic stewardship over our study period (S1 Fig).

The outcomes recorded and analysed were LOS, ICU admission at any time during that hospitalisation and 30-day all-cause mortality. The database was cross referenced with the national mortality database to ensure accuracy of mortality data. Approval by ethical committee was not sought as the data was collected as part of a collaborative quality improvement project at Salford Royal NHS Trust [11].

### Statistical analysis

Categorical data are presented as frequencies and percentages. Non-parametric data are presented with median and interquartile ranges. Chi square tests were used for between group comparisons of categorical data and Mann-U Whitney test for non-parametric continuous data. For the total population a binomial logistic regression model adjusted for age and sex was developed to analyse the associations between sCA-I and CA-AKI in stages and 30-day mortality, ICU admission and LOS dichotomised at median value for patients admitted to hospital. Results are presented as odds ratio (OR) with 95% confidence intervals (CI). The above analyses were repeated in the population subset resident in care homes. The care home population had very few ICU admissions therefore CA-AKI was not analysed by CA-AKI stage for this outcome in the regression model. An alpha value of < 0.05 was considered statistically significant. Analyses were performed in SPSS software (package version 23.0) under license to The University of Manchester.

## Results

### Patients, sCA-I and CA- AKI characteristics

During the study period there were 61,471 non-elective non-dialysis patient attendances to hospital who underwent phlebotomy for creatinine of which 2580 (4.2%) were from a care home. The median age at admission was 58 years old (interquartile range 33–77). 55.6% were female. In total 17,287 (28.1%) of attendees were prescribed antibiotics within 48hrs of attendance (sCA-I), 3,506 (5.7%) of attendees had CA-AKI (3.8% AKI1, 1.1% AKI2 and 0.8% AKI3). The antibiotics prescribed in sCA-I are displayed in S1 Table. 1443 (2.3%) attendees suffered CA-AKI alone and 2,063 (3.4%) had both sCA-I and CA-AKI (Table 1). Patient and sCA-I and CA-AKI characteristics based on source of admission (care home residents vs others) are presented in S2 Table.

**Table 1:** Length of stay, ICU and 30-day all-cause mortality grouped by presence or absence of sCA-I and CA-AKI. All patient attendances included (N = 61471).

	No CA-AKI or sCA-I N = 42740	sCA-I N = 15225	CA-AKI N = 1443	sCA-I and CA-AKI N = 2063
Age (years)	53 (30–75)	65 (40–79)	72 (56–82)	75 (63–84)
Male Sex	18355 (42.9%)	7250 (47.6%)	689 (52.3%)	1029 (49.9%)
Length of Stay (days)	1 (0–3)	3 (1–8)	4 (1–9)	7 (3–14)
ICU admission	593 (1.4%)	1063 (7%)	100 (6.9%)	457 (22.2%)
30-day all-cause mortality	1181 (2.8%)	1419 (9.3%)	184 (12.8%)	585 (28.4%)

Values expressed as N (%) or median (interquartile range). Abbreviations: sCA-I = Suspected community acquired infection, CA-AKI = Community acquired acute kidney injury, ICU = Intensive Care Unit. Length of stay data was available for 61,096 of attendances (99.4%)

### Outcomes

#### 30-day mortality

**Fig 1:**
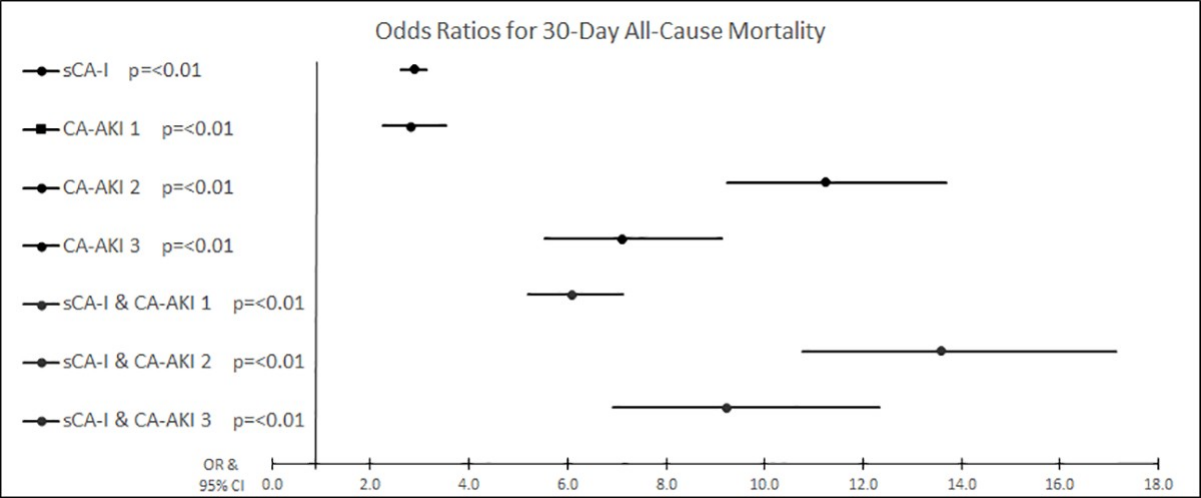
Forrest plot displaying the adjusted odds ratios for 30-day all-cause mortality in general population.

Thirty-day mortality for the whole population was 5.5% and was higher in patients with sCA-I (8.1%), those with CA-AKI (11.8%) and being greatest in patients with the combination of sCA-I and CA-AKI (26.2%). Within the care home population 30-day mortality for the group with both of sCA-I and CA-AKI was 43.4% (S2 Table). The binary logistic regression model for the general population demonstrated all variables were associated with 30-day mortality (Table 2, Fig 1). In general population sCA-I combined with CA-AKI stage 2 and 3 were associated with the highest mortality risk (Table 2, Fig 1). In care home residents the highest mortality risk was observed in the combination of AKI stage 3 and sCA-I (Table 3).

**Table 2:** Binary logistic regression model for 30-day all-cause mortality, ICU admission and LOS > 3 days for attendances from general population (N = 58891).

	30 Day Mortality			ICU Admission			LOS > 3 days		
	OR	CI	P value	OR	CI	P value	OR	CI	P value
**sCA-I**	2.88	2.63–3.14	<0.01	5.15	4.64–5.71	<0.01	4.31	4.12–4.51	<0.01
**CA-AKI** **(CA-AKI1 = 973, CA-AKI2 = 212, CA-AKI3 = 178)**									
**Stage 1**	2.82	2.26–3.52	<0.01	4.03	3.05–5.31	<0.01	2.47	2.15–2.84	<0.01
**Stage 2**	11.24	9.23–13.69	<0.01	18.80	15.21–23.23	<0.01	6.50	5.31–7.95	<0.01
**Stage 3**	7.12	5.55–9.14	<0.01	20.78	16.48–26.21	<0.01	8.26	6.49–10.50	<0.01
**Any Stage**	3.35	2.79–4.02	<0.01	4.79	3.82–6.00	<0.01	3.07	2.72–3.46	<0.01
**CA-AKI and sCA-I** **(CA-AKI1 = 1154, CA-AKI2 = 382, CA-AKI3 = 269,)**									
**Stage 1**	6.09	5.21–7.12	<0.01	12.52	10.54–14.88	<0.01	8.97	(7.62–10.56)	<0.01
**Stage 2**	13.58	10.77–17.13	<0.01	24.54	19.44–30.98	<0.01	7.61	5.84–9.91	<0.01
**Stage 3**	9.23	6.91–12.33	<0.01	29.26	22.46–38.18	<0.01	9.48	6.82–13.18	<0.01
**Any Stage**	7.79	6.87–8.83	<0.01	17.97	15.63–20.66	<0.01	8.72	7.67–9.92	<0.01

Abbreviations: LOS = length of stay, ICU–intensive care unit, sCA-I = Suspected community acquired infection, CA-AKI = Community acquired acute kidney injury, OR = Odds Ratio, CI = 95% Confidence interval. Patients residing in care homes were excluded from this analysis. Length of stay data was available for 61,096 of attendances (99.4%). All OR were adjusted for age and sex. Referent Group was no sCA-I and no CA-AKI

**Table 3:** Binary logistic regression model for 30-day all-cause mortality, ICU admission and LOS > 3 days for attendances from the care home population (N = 2580).

	30 Day Mortality			ICU Admission			LOS > 3 days		
	OR	CI	P value	OR	CI	P value	OR	CI	P value
**sCA-I**	3.31	2.62–4.12	<0.01	6.10	2.88–12.92	<0.01	4.17	3.48–4.99	<0.01
**CA-AKI** **(CA-AKI1 = 60, CA-AKI2 = 15, CA-AKI3 = 6)**									
**Stage 1**	3.01	1.62–5.59	<0.01				2.83	1.64–4.87	<0.01
**Stage 2**	2.47	1.45–4.21	<0.01				1.53	0.91–2.57	0.11
**Stage 3**	1.79	0.99–3.21	= 0.051				1.43	0.81–2.53	0.22
**Any stage**	3.65	2.16–6.16	<0.01	5.46	1.43–20.81	= 0.01	2.75	1.72–4.40	<0.01
**CA-AKI and sCA-I** **(CA-AKI1 = 149, CA-AKI2 = 75, CA-AKI3 = 32)**									
**Stage 1**	5.16	3.53–7.54	<0.01				8.04	5.04–12.83	<0.01
**Stage 2**	7.68	4.68–12.58	<0.01				7.03	3.82–12.93	<0.01
**Stage 3**	10.49	5.03–31.86	<0.01				6.22	2.54–15.22	<0.01
**Any Stage**	6.43	4.73–8.75	<0.01	11.37	5.50–23.51	<0.01	7.306	5.15–10.37	<0.01

Abbreviations: LOS = length of stay, ICU–intensive care unit, sCA-I = Suspected community acquired infection, CA-AKI = Community acquired acute kidney injury, OR = Odds Ratio, CI = 95% Confidence interval. All AKI stages were grouped together for LOS and ICU admissions. Length of stay data was available for 61,096 of attendances (99.4%). All OR were adjusted for age and sex. Referent group was no sCA-I and no CA-AKI

#### ICU admission

**Fig 2:**
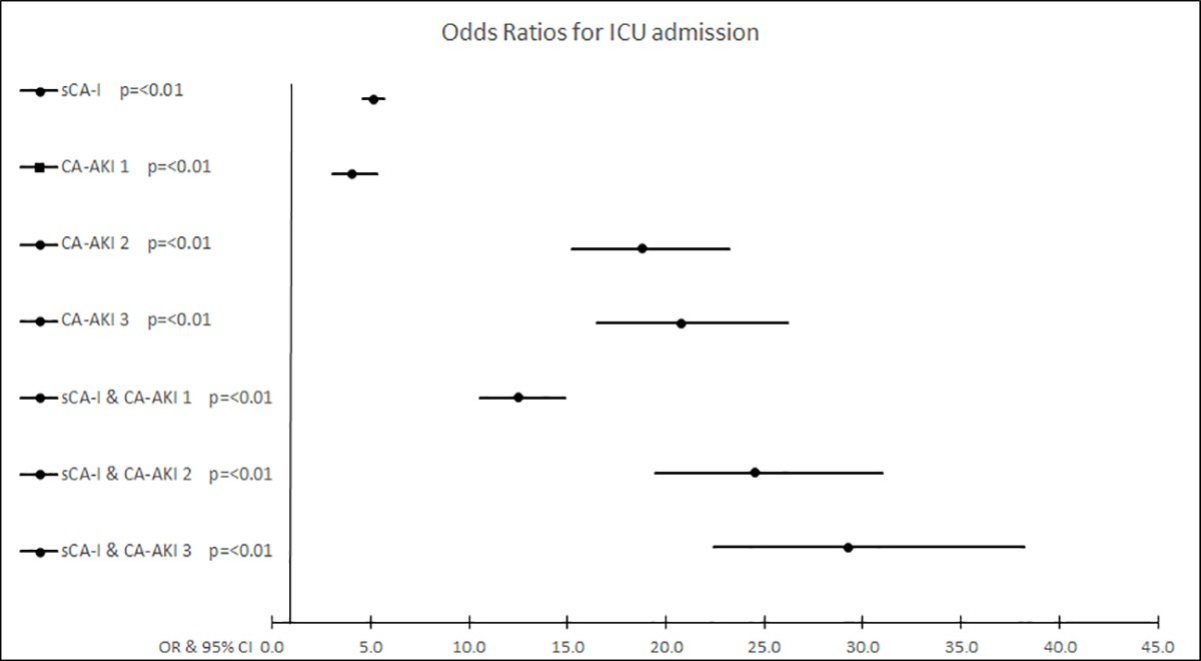
Forrest plot displaying the adjusted odds ratios for intensive care unit admission in general population.

In total there were 2213 (3.6%) patients admitted to ICU. The highest proportion of ICU admissions were by patients with both sCA-I and CA-AKI (24%), followed by sCA-I alone (7.2%) and CA-AKI alone (7.1%) (S2 Table). The greatest adjusted odds for ICU admission was observed in sCA-I combined with CA-AKI stage 3 (Table 2, Fig 2). Within the care home residents, the group with the highest proportion of ICU admissions was the combination of both sCA-I and CA-AKI (8.9%), (S2 Table). CA-AKI in combination with CA-I had the highest adjusted OR for ICU admission within the care home population compared with no sCA-I and no CA-AKI group (Table 3).

#### Length of stay

**Fig 3:**
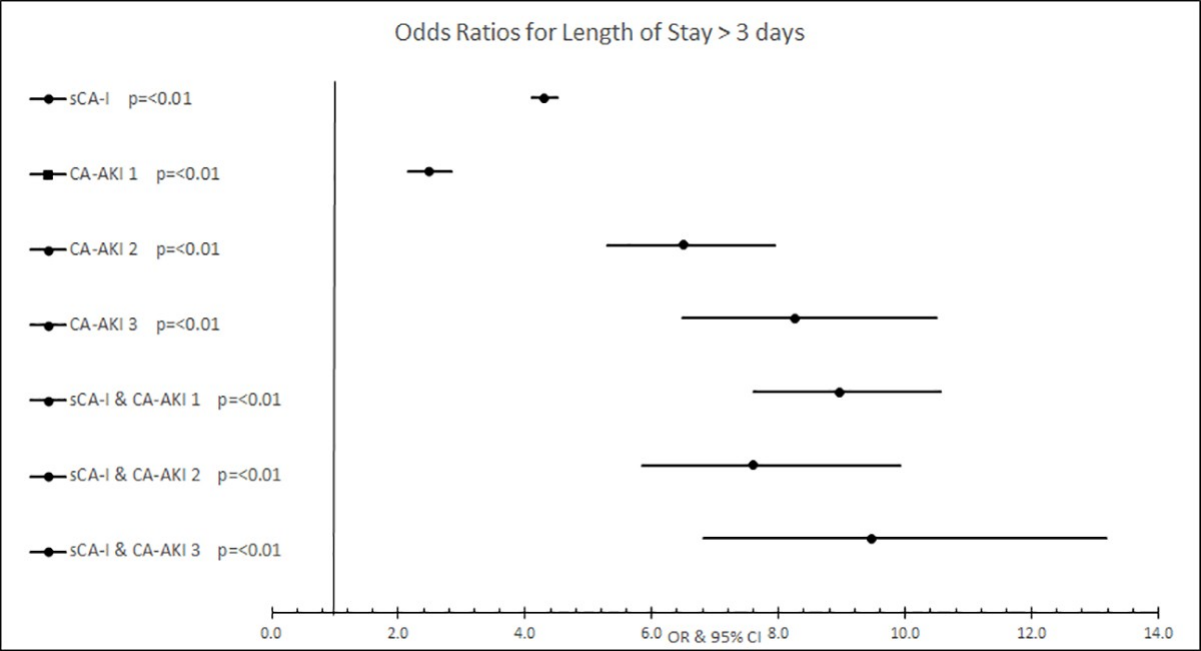
Forrest plot displaying the adjusted odds ratios for length of stay> 3 days in general population.

Length of stay data was available for 61,096 of attendances (99.4%). Length of stay was dichotomised at the median length of stay for admitted patients (3 days). 35.4% of general population patient attendances had a LOS more than 3 days compared with 58% of care home patient attendances. All model variables were significantly associated with LOS more than 3 days in general population. The highest risks were observed in the patients with the combination of sCA-I and CA-AKI, although there was little difference between AKI stages. Complete results are presented in Table 2 and displayed graphically in Fig 3. A similar finding was seen in care home population (Table 3).

## Discussion

This study has demonstrated that the combination of sCA-I and CA-AKI is present in 3.4% of non-elective hospital attendances and is associated with worse outcomes (30-day all-cause mortality, LOS and admission to ICU) than either CA-AKI or sCA-I on their own. The incidence of CA-AKI in this study (5.7%) and the 30-day mortality rate (21.9%) for the whole group is similar to other published studies [5,19]. In this study population the majority (58.8%) of CA-AKI cases were treated with antibiotics for suspected infection.

The finding that the presence of AKI in the context of suspected community acquired infection confers an increased mortality risk is in line with one previous study by Jain et al. [20]. This study used a coded diagnosis of AKI which occurred within 28 days of a primary care derived diagnosis of community acquired pneumonia between 2004 and 2011 and demonstrated that 8.4% of patients with community acquired pneumonia suffered from AKI. This study was performed prior to the implementation of AKI staging criteria and prior to increased awareness of AKI amongst healthcare professionals. In our study 11.9% of patient attendances with sCA-I also suffered CA-AKI.

Our results support the recommendation in the NICE guidance for the early recognition and management of sepsis that incorporates the presence of AKI as a risk stratification criterion to guide management of suspected infection in the hospital setting [21]. Furthermore, it is plausible that earlier detection of AKI in primary care during the evaluation of suspected infection may support clinical decision making in the evaluation and management of sepsis including prompt hospital referral in high risk cases. A UK study has shown that the majority of patients who attend the ED have also consulted their primary care provider first [22]. However, in primary care there is often a lag time between decision to take blood tests (if such decision is made) and phlebotomy and between phlebotomy and the result being reviewed, usually by another clinician and this lag may have an effect on outcomes in infections associated AKI and sepsis [23]. Improvement in time to response to AKI alerts has been associated with decreased mortality in AKI 2&3 alerting in primary care [24] and prompt delivery of the sepsis bundle has been shown to improve outcomes. The use of point of care creatinine testing may offer a useful adjunct to the assessment of suspected infection in the community has the potential to expedite delivery of the sepsis bundle in high risk patients and its utility should be prospectively explored.

In the general population and care home population there was no consistent increase in risk of 30-day mortality between CA-AKI2 and CA-AKI3 alone or in combination with sCA-I. This finding aligns with other studies which do not demonstrate a higher mortality for AKI3 compared to AKI2 [4]. There are some potential explanations for the lack of difference in mortality. First it is possible that this finding is due to misclassification of AKI stage by the algorithm. Within the AKI3 alert algorithm, in the absence of a baseline creatinine within the preceding week, the algorithm captures AKI3 cases using two rules. One of the rules alerts to AKI3 if the alerting creatinine value is over 354 μmol/L and ≥1.5 higher than the median value over the preceding 365 days. It is therefore liable to capture cases of chronic kidney disease (CKD) with relatively mild creatinine elevations. Second, it can be argued that AKI3, being the highest stage of AKI alert, may trigger different or more intensive management than AKI2 and thereby reduce mortality [6]. In our study patients with CA-AKI3 were more likely to be admitted to ICU than CA-AKI2 (24.7% v 21.3%). Finally, we only captured data over a 48-hour time window from admission and it is possible that some cases of CA-AKI2 were progressing to AKI3. Based on our results CA-AKI stages 2 and 3 should be considered as conferring similar risk.

To our knowledge this is the first study to examine the utility of the NHS AKI e-alert and outcomes of suspected infection and CA-AKI in a large cohort of care home residents. In this vulnerable population 13.1% of patients who attended hospital suffered CA-AKI, more than double the incidence of CA-AKI in the general population. The care home population has demonstrated similar increasing associations of poor outcomes with sCA-I, CA-AKI and the combination of the two conditions. Combination of sCA-I and CA-AKI was associated with extremely high 30-day all-cause mortality at 43.4%.

There are some limitations to this research. We have described suspected CA-I and not definitive infection based on positive cultures or infection markers and therefore it is possible that antibiotics were prescribed inappropriately in some of these cases. This study’s aim was to ascertain the impact of the clinical suspicion of CA-I alone and in combination with CA-AKI and not definitive infection and therefore a degree of caution should be exercised in the interpretation of our findings. Physiological parameters included in the qSOFA score were not used in our analysis because we could not retrieve this data with accuracy. Patients from care homes have reduced muscle mass and therefore AKI criteria may work differently and underestimate AKI severity in this group. This dataset did not include urine output, other comorbidities and risk factors for AKI and death such as circulatory disease, malignancy, use of immunosuppression or potentially nephrotoxic medications therefore these factors could not be included in regression models. Patients with CA-AKI and a haemodialysis or hemofiltration procedure performed during that admission were re-coded as CA-AKI3 although it was not possible to prove retrospectively that renal replacement had occurred within 48hours of admission. Patients who did not trigger CA-AKI but were started on renal replacement therapy were not included because it was not possible to determine the indication for renal replacement and whether the therapy had commenced within 48hours of admission. The addition of sCA-I to CA-AKI made a dramatic increase to the likelihood of ICU admission. It is difficult to determine the direction of causality in this circumstance because patients admitted to ICU are inherently more unwell, undergo more invasive procedures and antibiotic treatments are often empirically prescribed. A further limitation is that this study only investigated outcomes up to 30 days from the event. Wonnacott et al [8] demonstrated a surprisingly high long term mortality rate with CA-AKI. The interaction between sCA-I and CA-AKI in relation to long term mortality was not investigated in this study. Finally, this large single centre UK study’s outcomes are only translatable to communities with similar health care systems. Outcomes of CA-AKI and CA-I in less developed countries are likely to be different.

## Conclusion

This study demonstrated that there is an increasing association for poor outcomes with sCA-I, CA-AKI and both sCA-I and CA-AKI in combination. The aggregation of sCA-I has a detrimental effect on patient outcomes if they are suffering CA-AKI and vice versa. This study confirms the importance of AKI when infection is considered. The potential role of early assessment for AKI in suspected infection during triage in the emergency department and in community settings and care homes using point of care testing should be prospectively investigated.

## Supporting information

S1 Table The antibiotics prescribed in patients who were admitted with suspected community acquired infection. (DOCX)

S2 Table Demographics and outcomes for the hospital attendances separated by the presence of absence of CA-AKI or suspected CA-I and source of admission. (DOCX)

S1 Fig A graph demonstrating monthly compliance with antibiotic prescriptions during the study period and compliance audit criteria. (DOCX)

S1 Dataset Anonymised data used in this study. (XLSX)

## Abbreviations

ESRD: End stage renal disease
AKI: Acute kidney injury
sCA-I: Suspected community acquired infection
CA-AKI: Community acquired acute kidney injury
OR: Odds ratio
LOS: Length of stay
ICU: Intensive care unit
NHS: National health service
HA-AKI: Hospital acquired acute kidney injury
KDIGO: Kidney disease improving global outcomes
CKD: Chronic kidney disease
CI: Confidence interval
qSOFA: Quick sequential organ failure assessment
