# Supplementary material for: The importance of acute kidney injury in suspected community acquired infection
Source: PLoS One. 2019 May 7;14(5):e0216412. doi: 10.1371/journal.pone.0216412 (PMC6504101; doi:10.1371/journal.pone.0216412)
Supplement: S1 Table — (DOCX) [file pone.0216412.s001.docx]

**Table S1:** The antibiotics prescribed in patients who were admitted with suspected community acquired infection

| Amoxicillin | 1873 |
| --- | --- |
| Benzylpenicillin | 2551 |
| Cefotaxime | 232 |
| Ceftazidime | 3 |
| Ceftriaxone | 893 |
| Cefuroxime | 46 |
| Chloramphenicol | 13 |
| Ciprofloxacin | 503 |
| Clarithromycin | 1381 |
| Clindamycin | 156 |
| Co-Amoxiclav | 1351 |
| Co-Trimoxazole | 98 |
| Colistimethate | 1 |
| Daptomycin | 8 |
| Doxycycline | 10 |
| Ertapenem | 21 |
| Flucloxacillin | 1551 |
| Fosfomycin | 6 |
| Gentamicin | 1709 |
| Levofloxacin | 30 |
| Linezolid | 6 |
| Meropenem | 265 |
| Metronidazole | 378 |
| Moxifloxacin | 2 |
| Piperacillin & Tazobactam | 3869 |
| Rifampicin | 10 |
| Teicoplanin | 52 |
| Tigecycline | 1 |
| Vancomycin | 268 |
| **Total** | **17287** |
