# Supplementary material for: The importance of acute kidney injury in suspected community acquired infection
Source: PLoS One. 2019 May 7;14(5):e0216412. doi: 10.1371/journal.pone.0216412 (PMC6504101; doi:10.1371/journal.pone.0216412)
Supplement: S2 Table — (DOCX) [file pone.0216412.s002.docx]

**Table S2.** Demographics and outcomes for the hospital attendances separated by the presence of absence of CA-AKI or suspected CA-I and source of admission.

|  | No sCA-AKI or CA-I | | sCA-I | | CA-AKI | | sCA-I and CA-AKI | |
| --- | --- | --- | --- | --- | --- | --- | --- | --- |
|  | General Population | Care Home | General Population | Care Home | General Population | Care Home | General Population | Care Home |
| Total Attendances | 41522 | 1218 | 14202 | 1023 | 1362 | 81 | 1805 | 258 |
| Age (years) | 52 (30-74) | 85 (77-90) | 62 (37-77) | 85 (78-90) | 71 (55-81) | 84 (78-89) | 74 (62-82) | 85 (77-89) |
| Male (%) | 17930  (43.2) | 425  (34.9) | 6834 (48.1) | 416 (40.7) | 659 (48.4) | 30 (37.0) | 920 (51.0) | 109 (42.2) |
| ICU Admission (%) | 584 (1.4) | 9 (7) | 1025 (7.2) | 38 (3.7) | 97 (7.1) | 3 (3.7) | 434 (24.0) | 23 (8.9) |
| LOS >3days (%) | 10362 (25.1) | 479 (39.6) | 8105 (57.5) | 742 (73.0) | 801 (59.5) | 52 (64.2) | 1459 (81.8) | 212 (83.5) |
| 30-day all-cause mortality (%) | 1060 (2.6) | 121 (9.9) | 1147 (8.1) | 272 (26.6) | 161 (11.8) | 23 (28.4) | 473 (26.2) | 112 (43.4) |

^Abbreviations: LOS = length of stay, ICU – intensive care unit, sCA-I = Suspected community acquired infection, CA-AKI= Community acquired acute kidney injury, Length of stay data was available for 61,096 of attendances (99.4%)^
