# Supplementary material for: The importance of acute kidney injury in suspected community acquired infection
Source: PLoS One. 2019 May 7;14(5):e0216412. doi: 10.1371/journal.pone.0216412 (PMC6504101; doi:10.1371/journal.pone.0216412)
Supplement: S1 Fig — (DOCX) [file pone.0216412.s003.docx]

**Supplementary Material**

**Fig S1.** A graph demonstrating monthly compliance with antibiotic prescriptions during the study period and compliance audit criteria.

Audit criteria for compliance included:

1) The indication/rationale for antimicrobial therapy is documented, including clinical criteria relevant to this.

2) The antibiotic choice is in line with antibiotic guidelines and any clinical criteria relevant to the choice of agent are documented (e.g. CURB-65, severity criteria)

3) A management plan is documented, including an appropriate stop or review date

4) Microbiology results are reviewed and consideration is given to stepping down to focussed (narrow spectrum) antibiotic therapy

5) Where appropriate, IV to oral switch is undertaken after 24-48 hours in line with the antibiotic guidelines
